# The use of heart rate variability measures as indicators of autonomic nervous modulation must be careful in patients after orthotopic heart transplantation

**DOI:** 10.1007/s10877-015-9747-y

**Published:** 2015-08-14

**Authors:** Wan-An Lu, Gau-Yang Chen, Chun-Che Shih, Cheng-Deng Kuo

**Affiliations:** 1 Laboratory of Biophysics, Department of Medical Research, Taipei Veterans General Hospital, Taipei, 112 Taiwan; 2 Division of Respiratory Therapy, Department of Chest Medicine, Taipei Veterans General Hospital, Taipei, 112 Taiwan; 3 Institute of Cultural Asset and Reinvention, Fo-Guang University, Yilan, 262 Taiwan; 4 Department of Internal Medicine, Ten-Chen General Hospital, Yangmei, Tao-Yuan, 326 Taiwan; 5 Institute of Biomedical Engineering, National Yang-Ming University, Taipei, 112 Taiwan; 6 Division of Cardiovascular Surgery, Department of Surgery, Taipei Veterans General Hospital, Taipei, 112 Taiwan

**Keywords:** Autonomic nervous system, Heart transplantation, Heart rate variability, Re-innervation

## Abstract

The precise relation between heart rate variability (HRV) and autonomic re-innervation has not been established explicitly in patients after orthotopic heart transplantation (OHT), but can be inferred from the fact that the HRV is reduced immediately after OHT and may increase gradually with time. The aim of this study was to investigate the residual HRV in patients about 1–2 years after OHT, as compared with patients after coronary artery bypass graft (CABG) surgery. Thirteen patients who had received OHT and 14 patients who had received CABG surgery were recruited. HRV analysis was performed and the HRV measures in supine position were compared between these two groups of patients. We found that the mean (mRRI), standard deviation and coefficient of variation of RR intervals, total power, very low frequency power (VLFP), low frequency power, high frequency power (HFP), normalized VLFP (nVLFP) and low-/high-frequency power ratio in the OHT group were all significantly decreased, while the heart rate (HR) and normalized HFP (nHFP) were significantly increased, as compared with the CABG group. The decrease in HRV was more severe in the VLFP region. A smaller nVLFP and a greater nHFP were associated with a smaller mRRI and a larger HR in the OHT patients. The slope of the power law relation of HRV became positive in OHT patients, instead of negative in CABG patients. We conclude that patients after OHT have residual HRV which were characterized by severely depressed time and frequency domain HRV, increased HR and nHFP, decreased nVLFP, and positive slope of the power-law relation of HRV. The use of nHFP as the indicator of vagal modulation and the use of nVLFP as the indicator of renin-angiotensin modulation, thermoregulation and vagal withdrawal must be careful in the OHT patients.

## Introduction

Spontaneous beat-to-beat fluctuation in heart rate (HR) reflects the ongoing modulation of sinus node activity through several cardiovascular control mechanisms [1]. This fluctuation in heart rate is termed heart rate variability (HRV), and can be quantified by the mathematical technique of power spectral analysis, which calculates the frequency contents of the time-varying signals and plots the power of the frequency bands as a function of frequency. The spectrum obtained from power spectral analysis of sequences of RR intervals (RRI) can be divided mainly into two parts, a low frequency (LF) component and a high frequency (HF) component. The LF component is thought to be related to both sympathetic and vagal innervations, while the HF component is thought to be related to the vagal innervation alone on the basis of experiments involving specific intravenous autonomic blocking agents [2–4]. Below the LF range, a very-low frequency range and an ultra-low frequency range have been suggested. The very-low frequency range reflects the renin-angiotensin-aldosterone modulation and vagal withdrawal [5–7], and the ultra-low frequency range is often used in the power law relationship [8]. The HRV measures can be used as non-invasive prognostic indices of the autonomic nervous modulation of the subjects with many kinds of diseases [9–12]. For instance, reduced cardiac vagal modulation has been reported in patients with coronary artery disease (CAD) [13, 14], and the reduction in the cardiac vagal modulation was found to correlate with the angiographic severity, independent of previous myocardial infarction, location of diseased coronary arteries, and left ventricular function [15].

Although the re-innervation might be inferred from the HRV analysis, the real time course of change in HRV after orthotopic heart transplantation (OHT) has not been fully established yet. Partial re-innervation of both sympathetic and vagal branches has been reported after OHT in dogs [16–18]. The cardiac denervation associated with OHT and subsequent potential for re-innervation have been the focus of interest among clinicians, physiologists, and pharmacologists [19]. In a study using 24-h Holter recordings performed on 37 ambulant patients 1–122 months after cardiac transplantation, patients >36 months after transplant had lower 24-h HR, an increased average of all 5-min SDs of NN intervals, and higher low-frequency power (LFP) and high-frequency power (HFP), suggesting that patients late after cardiac transplantation have HRV evidence for an increase in sympathetic control of the heart [20]. In another 10-year longitudinal follow-up study, the observed increase in total power (TP), absolute LFP and HFP, but unchanged relative LFP and HFP after heart transplantation are compatible with partial re-innervation of the cardiac sinus node [21]. Thus, whether re-innervation has occurred and to what extent has re-innervation occurred is a matter of uncertainty.

We hypothesized that unless the re-innervation is nearly complete, the HRV measures may not be regarded as the true indices of autonomic nervous modulation of the OHT patients. Thus, the aim of this study was to examine the residual HRV in patients 1–2 years after OHT to see if their HRV measures can be used as the indices of autonomic nervous modulation.

## Methods

### Study design

This was a prospective case–control study. The Institutional Review Board (responsible person: Dr. Fa-Yauh Lee) of Taipei Veterans General Hospital, a tertiary medical center in Taiwan, has approved this study, and a written informed consent was obtained from each subject before the study. Patients who had received OHT because of dilated cardiomyopathy or ischemic cardiomyopathy about 1–2 years before the HRV study were recruited as the study group. At the time of HRV study, the post-transplantation follow-up period in the OHT group was 16.5 (median) (IQR 15.3–17.0) months, ranging from 13 to 24 months. If clinical condition allowed, no autonomic blocking agent was used on the patients for at least 24 h prior to the study. For ethical reason, patients who had signs or symptoms of active cardiorespiratory distress were excluded from this study. Electrocardiographic (ECG) signals and relevant clinical and laboratory data were collected during the scheduled routine follow-up visits.

Patients about 1–2 years after coronary artery bypass graft (CABG) surgery were recruited as the control group. Cardiopulmonary bypass was accomplished with single venous and single arterial cannulation at the ascending aorta. The exclusion criteria included atrial fibrillation, myocardial infarction within 6 months, or using class I antiarrhythmic medication. The CABG patients were recruited as the control group because they had received open chest surgery as the OHT patients did, but did not have their cardiac autonomic nervous innervation injured as the OHT patients did. In addition, some OHT patients had underlying ischemic heart disease before surgery, similar to the CABG patients. Thus, the CABG patients were chosen as the control group in this study.

### HRV analysis

All subjects were instructed not to drink caffeinated beverages for at least 24 h prior to ECG recording. After 5 min rest in supine position, a trend of ECG signals was picked up by a bedside ECG monitor (Biochem Vital Sign Monitor, BCI International, Waukesha, Wisconsin, USA), and was transmitted to a personal computer for recording for 15 min. During the rest and recording periods, the patient was asked to close his/her eyes to avoid visual interference from the environment. The sampling frequency for ECG recording was 500 Hz.

The recorded ECG signals were retrieved afterwards to measure the consecutive RRI by using the software for the detection of R waves. Sinus pauses and atrial or ventricular arrhythmia were deleted, and the last 512 stationary RRI were obtained for HRV analysis. If the percentage of deletion was >5 %, then the patient was excluded from statistical analysis.

The mean (mRRI), standard deviation (SD_RR_) and coefficient of variation (CV_RR_) of 512 stationary RRI were calculated using standard formulae for each subject. The power spectra of RRI were obtained by means of fast Fourier transformation (Mathcad 11, Mathsoft Inc., Cambridge, MA, USA). Direct current component was excluded before the calculation of the power. The area-under-the-curve of the spectral peaks within the range of 0.01–0.4, 0.01–0.04, 0.04–0.15, 0.15–0.40 Hz were defined as the TP, very low-frequency power (VLFP), LFP, and HFP, respectively. The normalized VLFP (nVLFP = VLFP/TP), normalized LFP (nLFP = LFP/TP), normalized HFP (nHFP = HFP/TP), and low-/high-frequency power ratio or low-/high- ratio in short (LHR = LFP/HFP) were used as the indices of renin-angiotensin modulation, thermoregulation and vagal withdrawal, combined sympathetic and vagal modulation, vagal modulation, and sympatho-vagal balance, respectively [22].

The HRV is known to be affected by a complex interplay of neural, humoral, and electrophysiological factors, which in turn are modulated by central and peripheral oscillators [23]. In order to evaluate the complexity of the controlling system of HR in patients after OHT, the power-law characteristics of HRV was obtained by plotting the logarithm of power against the logarithm of frequency to obtain a regression line that can be described by three parameters, i.e., the Pearson correlation coefficient ρ, slope, and Y-intercept [24–29]. Briefly, the linear regression analysis between log(power) and log(frequency) within the frequency range of 0.01 and 0.5 Hz was performed by least-square method to obtain the three parameters of best fit regression line.

### Statistical analysis

Mann–Whitney rank sum test (SigmaStat statistical software, SPSS Inc., Chicago, Illinois, USA) was employed to compare the baseline characteristics and the HRV measures between OHT and CABG groups. Chi-square or Fisher exact test when appropriate was employed to compare the categorical data of the baseline characteristics between OHT and CABG groups. The correlation between the time interval between OHT surgery and HRV analysis, and between HRV measures and power-frequency relation characteristics in patients after CABG or OHT surgery were assessed by Pearson product moment correlation analysis. The data are presented as medians and interquartile range (25–75 %). A *P* < 0.05 was considered statistically significant.

## Results

### Clinical and hemodynamic characteristics

Thirteen patients after OHT surgery were included in the OHT group. At the time of surgery, the recipients’ age was 55.0 (43.5–66.0) years, ranging from 34 to 75 years. All patients in the OHT group were receiving immunosuppressive therapy. Endomyocardial biopsy did not show evidence of tissue rejection in all OHT patients at the time of study. Nine OHT patients were classified as NYHA class 2, and four patients were classified as NYHA class 3. Five patients in the OHT group were transplanted due to dilated cardiomyopathy, seven patients due to ischemic cardiomyopathy, and one patient due to rheumatic heart disease with severe mitral stenosis and regurgitation.

Fourteen patients 1–2 year after CABG surgery were recruited as the control group. A mean of 4.1 grafts per patient was anastomosed using saphenous vein and internal mammary artery. All patients improved symptomatically after CABG surgery during the follow-up period. There were no neurological symptoms or signs after CABG in these patients. The CABG patients were classified as NYHA class 1–2 when HRV analysis was performed.

Table 1 shows the basic and hemodynamic data of the patients in the OHT and CABG groups. Diabetes mellitus was defined as fasting blood glucose 126 mg/dl or higher. Hypertension was defined as systolic blood pressure >140 mmHg or diastolic blood pressure >90 mmHg. Hyperlipidemia was defined as total cholesterol >200 mg/dl or low density lipoprotein cholesterol >100 mg/dl. All patients in the CABG group had coronary artery disease (CAD) with 2 or 3 vessels diseases, and 12 out of 13 patients in the OHT group had cardiomyopathy. That’s the reason why they received CABG or OHT surgery, respectively. Most patients in the CABG group were using nitrates, while most patients in the OHT group were using immunosuppressants and prednisolone.

**Table 1:** Baseline characteristics of patients in the CABG and OHT groups

	CABG group (n = 14)	OHT group (n = 13)	*P* value
Age (years)	63.5 (55.5-66.3)	56.0 (44.5-67.0)	0.319
Gender (M/F)	12/2	12/1	1.000
Body height (cm)	162 (157–168)	165 (162–170)	0.230
Body weight (kg)	64 (57–80)	64 (59–72)	0.923
BMI (kg/m^2^)	25.1 (22.1–27.0)	22.9 (21.6-27.3)	0.790
Post-OHT period (month)	–	16.5 (15.3-17.0)	
History			
Previous MI	5	1	0.198
Cardiomyopathy	0	12	<0.001
CAD	14	3	<0.001
Hypertension	11	10	0.719
Diabetes mellitus	6	8	0.558
Hyperlipidemia	4	5	0.892
Medication			
Beta-blocker	5	1	0.198
Calcium antagonist	9	13	0.059
Nitrates	12	0	<0.001
ACE inhibitor	6	4	0.802
Digitalis	2	0	0.496
Aspirin	10	9	0.767
Immunosuppressants	0	13	<0.001
Prednisolone	0	13	<0.001
Clinical status			
LVEDP (mmHg)	14.5 (13.0–17.0)	–	
LVEF (%)	48.5 (45.0–53.0)	–	
NYHA functional class	2.0 (2.0–3.0)	–	
Two-vessel disease	2	2	0.644
Three-vessel disease	12	1	<0.001
Left main disease	4	1	0.368
LV aneurysm	3	0	0.247

Values are presented as medians (25–75 %)

*OHT* orthotopic heart transplantation, *BMI* body mass index, *ACE* angiotensin-converting enzyme, *LV* left ventricle, *LVEDP* left ventricular end-diastolic pressure, *LVEF* left ventricular ejection fraction, *MI* myocardial infarction, *CAD* coronary artery disease, *NYHA* New York Heart Association

### HRV after OHT and CABG

**Fig. 1:**
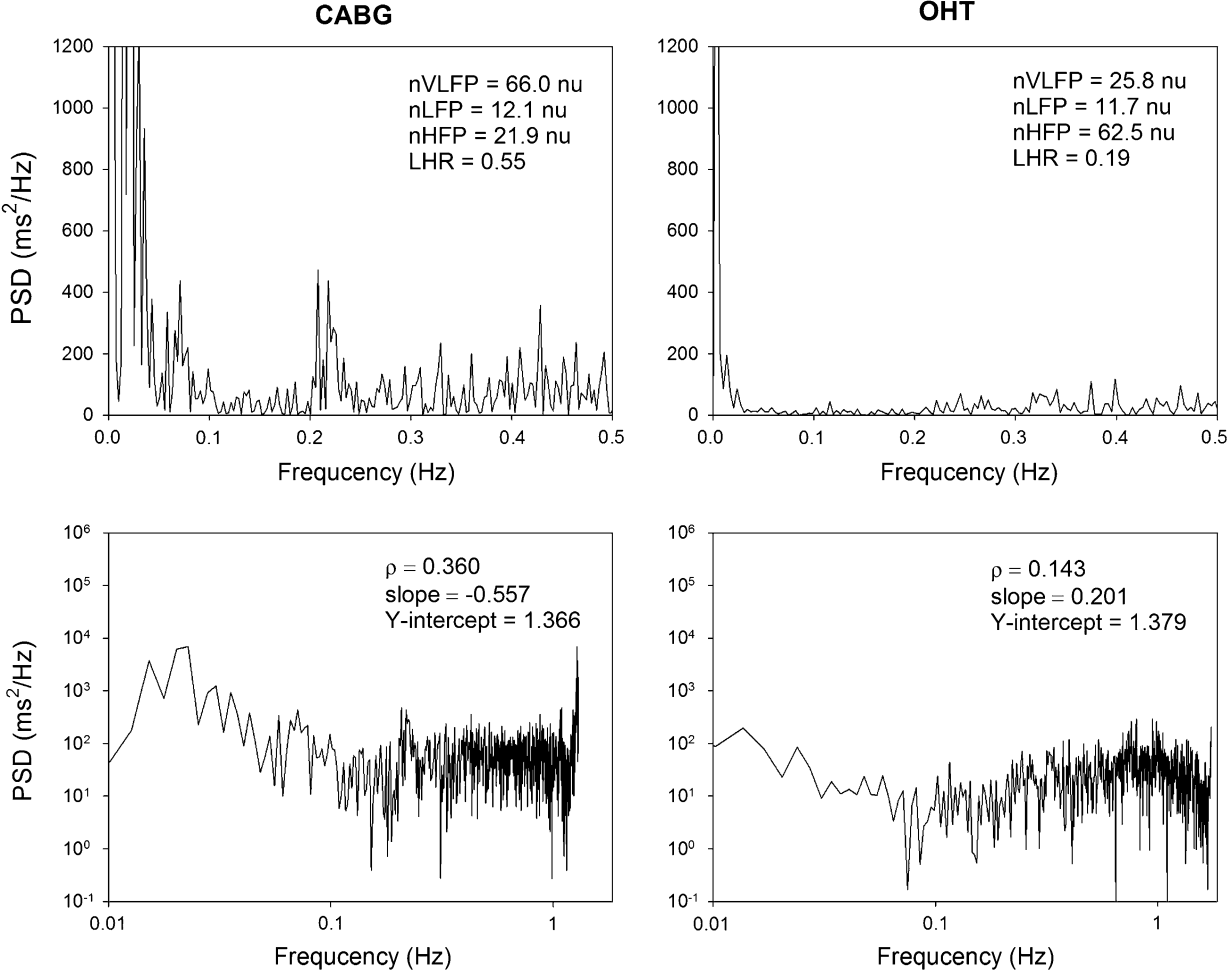
Representative power spectra of HRV in supine position in a patient in the CABG group and a patient in the OHT group. *nu* normalized unit, *PSD* power spectral density

Figure 1 show the representative power spectra and power law relation of HRV in supine positions in a patient in the CABG group and a patient in the OHT group, respectively. While the nVLFP and LHR of the OHT patient were lower than those of the CABG patient, the nHFP of the OHT patient was higher than that of the CABG patient. Similarly, while the slope of the power-law characteristics of HRV was negative in the CABG group, it became positive in the OHT group.

**Fig. 2:**
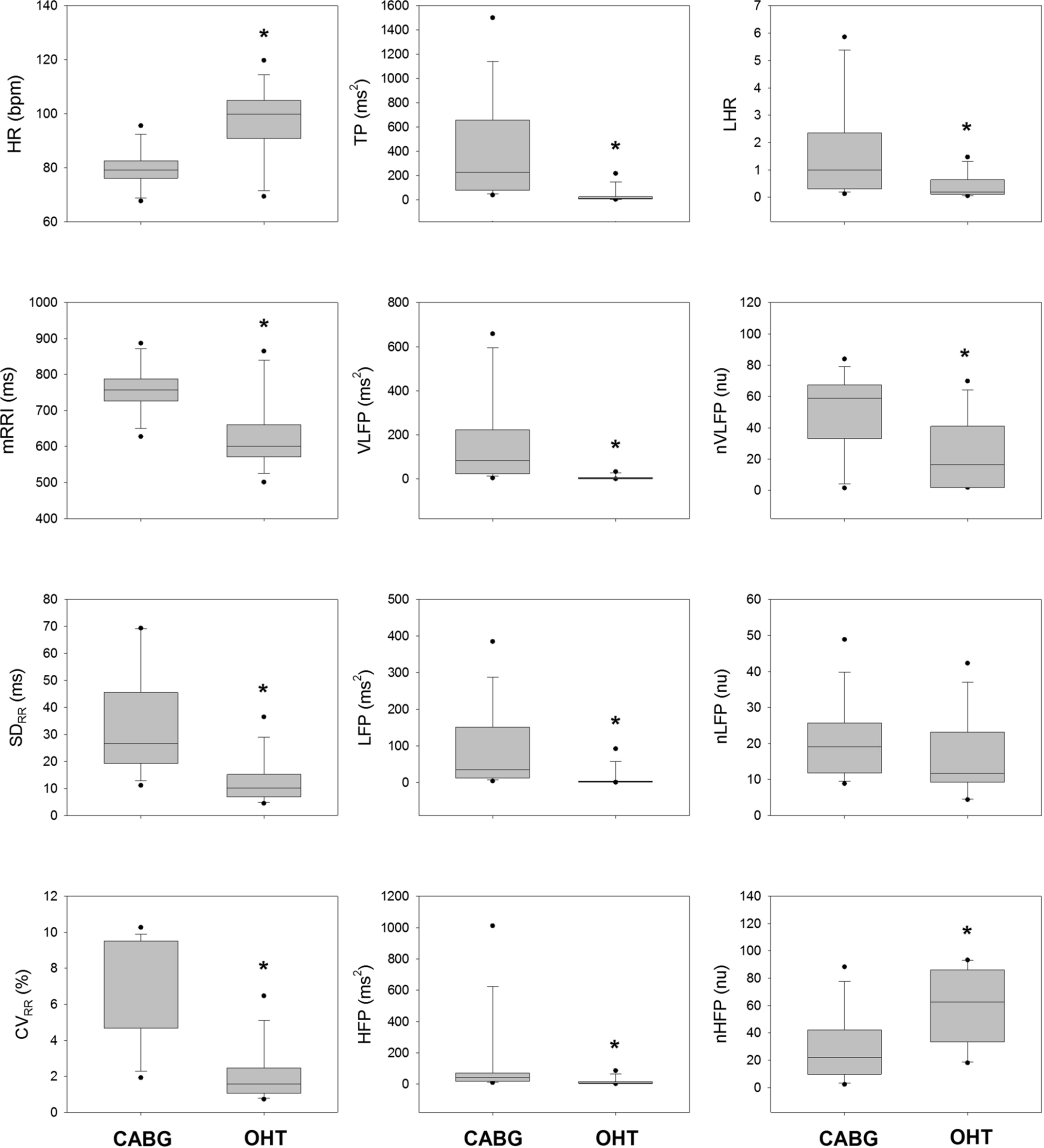
Comparison of HRV measures between CABG and OHT groups. *P < 0.05 versus CABG (Mann–Whitney rank sum test). While the HR was significantly greater, the mRRI, SD_RR_ and CV_RR_ of the OHT patients were all significantly smaller than those of the CABG patients. The TP, VLFP, LFP, HFP, nVLFP, LHR of the OHT patients were all smaller than those of the CABG patients, while the nHFP of the OHT patients was higher than that of the CABG patients

The left panels of Fig. 2 compares the time-domain HRV measures between the CABG group and the OHT group. While the HR was significantly greater, the mRRI, SD_RR_ and CV_RR_ of the OHT patients were all significantly smaller than those of CABG patients. The middle and right panels of Fig. 2 compare the frequency-domain HRV measures between CABG and OHT groups. The TP, VLFP, LFP, HFP, nVLFP, and LHR of the OHT patients were all significantly smaller than those of the CABG patients. In contrast, the nHFP of the OHT patients was significantly greater than that of the CABG patients.

**Fig. 3:**
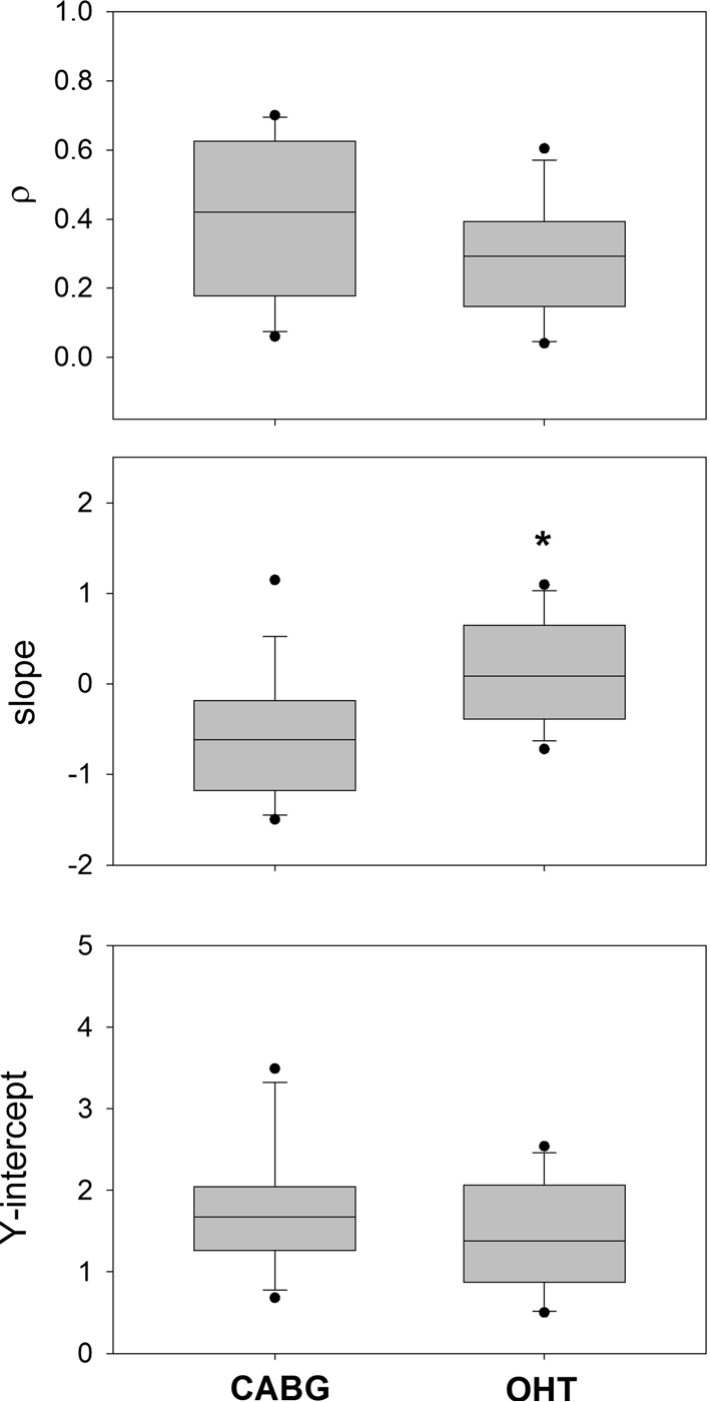
Comparison of power law characteristics of HRV between CABG and OHT groups. *P < 0.05 versus CABG (Mann–Whitney rank sum test)

Figure 3 shows the comparison of the characteristics of the power-frequency relationship between two groups of patients. Though there were no significant differences in the correlation coefficient and the Y-intercept between the CABG and OHT groups, the slope was significantly different between these two groups of patients. It is noteworthy that while the slope was negative in the CABG group, it became positive in the OHT group.

### Correlation analysis

**Fig. 4:**
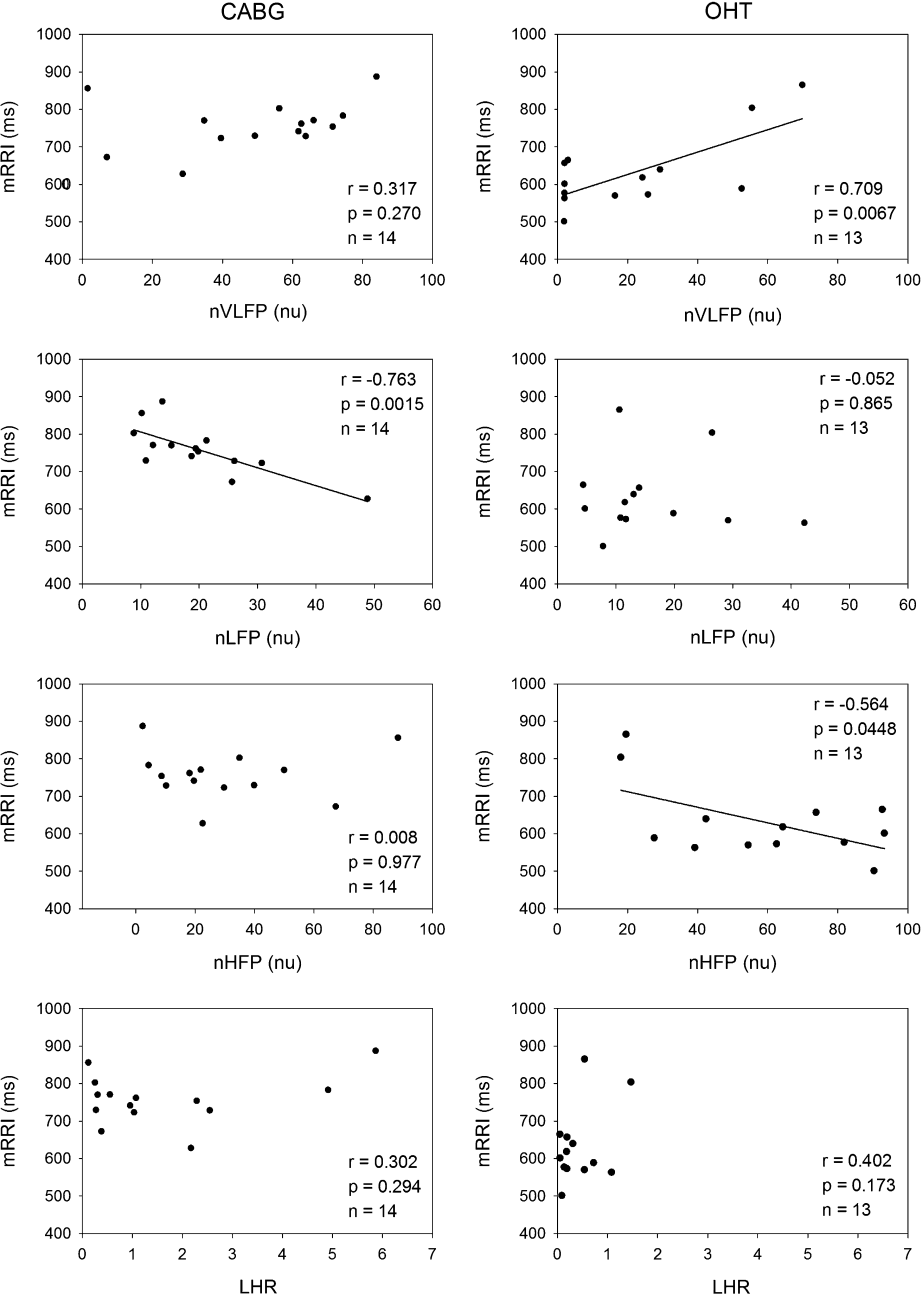
Linear correlations between mRRI and normalized HRV measures in the CABG and OHT patients. The mRRI correlates significantly and negatively with nLFP in CABG patients, but not OHT patients. On the other hand, the mRRI correlates significantly and positively with nVLFP and significantly and negatively with nHFP in OHT patients, but not CABG patients. *mean RRI* mean RR interval, *nVLFP* normalized very low-frequency power, *nLFP* normalized low-frequency power, *nHFP* normalized high-frequency power, *LHR* low-/high- frequency power ratio

Figure 4 shows that the mRRI correlated significantly and negatively with nLFP in the CABG patients, but not in the OHT patients. In contrast, the mRRI correlated significantly and positively with nVLFP and significantly and negatively with nHFP in the OHT patients, but not in the CABG patients. Thus, a smaller nVLFP and a greater nHFP were associated with a smaller mRRI in the OHT patients.

**Fig. 5:**
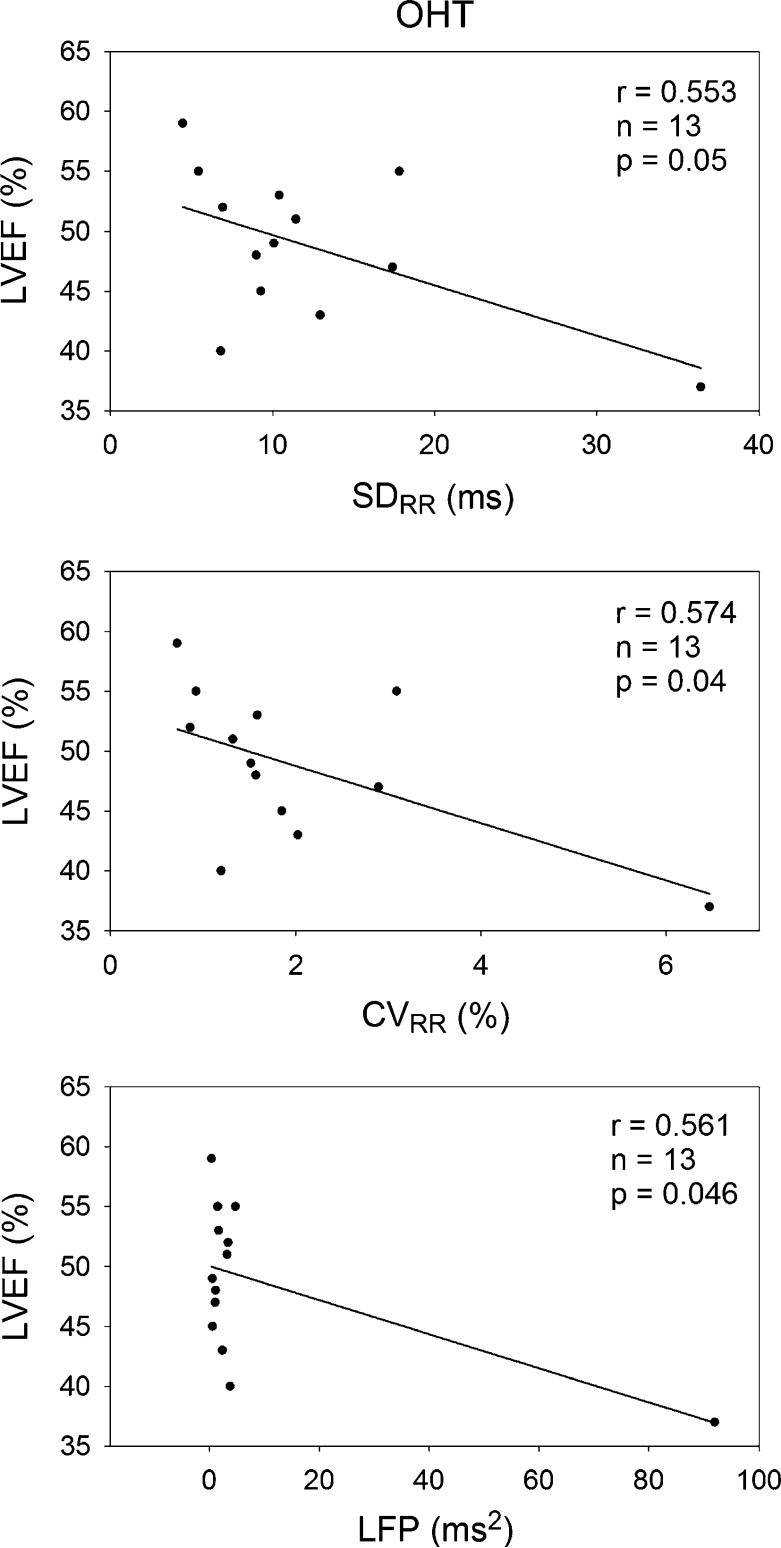
Significant correlations between left ventricular ejection fraction (LVEF) and HRV measures in the OHT group. The LVEF correlates significantly and negatively with SD_RR_, CV_RR_, and LFP in the OHT patients. *SD*_*RR*_ standard deviation of RR intervals, *CV*_*RR*_ coefficient of variation of RR intervals, *LFP* low-frequency power

Figure 5 shows that the LVEF correlated significantly and negatively with SD_RR_, CV_RR_, and LFP in the OHT group. A larger SD_RR_, CV_RR_, and LFP were associated with a smaller LVEF. In other words, an increase in global HRV or combined sympathetic and vagal modulation was associated with a decrease in LV function, if the LFP could still be regarded as an index of combined vagal and sympathetic modulation of the patients 1–2 years after OHT surgery.

Table 2 shows that the slope of linear regression analysis between log(power) and log(frequency) within the frequency range of 0.01 and 0.5 Hz correlated significantly and positively with nHFP, and correlated significantly and negatively with nVLFP and LHR in both CABG and OHT groups. An additional significant and negative correlation was found between the slope and nLFP in the OHT group, but not in the CABG group.

**Table 2:** Correlation analysis between the HRV measures and the characteristics of power-frequency relation of HRV in the CABG and OHT groups

	*ρ*	Slope	Y-intercept
CABG group			
nVLFP (nu)	0.436 (0.12)	−0.824 (<0.001)	−0.900 (<0.001)
nLFP (nu)	0.071 (0.81)	−0.235 (0.42)	0.083 (0.78)
nHFP (nu)	−0.467 (0.09)	0.926 (<0.001)	0.866 (<0.001)
LHR	0.686 (<0.01)	−0.719 (<0.01)	−0.524 (0.05)
OHT group			
nVLFP (nu)	−0.069 (0.82)	−0.785 (<0.01)	−0.794 (<0.01)
nLFP (nu)	−0.393 (0.18)	−0.616 (0.03)	−0.064 (0.84)
nHFP (nu)	0.314 (0.30)	0.949 (<0.001)	0.614 (0.03)
LHR	−0.107 (0.73)	−0.762 (<0.01)	−0.361 (0.23)

Values are correlation coefficients (*P* value)

*ρ* Pearson correlation coefficient, *slope* the slope of linear correlation between log(power) and log(frequency), *nVLFP* normalized very low frequency power, *nLFP* normalized low-frequency power, *nHFP* normalized high-frequency power, *LHR* low-/high-frequency power ratio

There were no significant correlations between the time interval after OHT surgery and the HRV measures in the OHT group (data not shown).

## Discussion

By using HRV analysis, we found that the patients after OHT had smaller time and frequency domain HRV measures than those of the patients after CABG, except that the HR and nHFP of the OHT patients were greater than those of CABG patients. The depression in various frequency domain HRV measures was more severe in the VLF range. A greater nVLFP is associated with a larger mRRI in the OHT patients. In contrast to the CABG patients and other kinds of patients, the slope of the power-law relation of HRV between log(power) and log(frequency) became positive in the patients after OHT.

In this study, the increase in both HR and nHFP and a simultaneous decrease in LHR suggested that the use of nHFP as the index of vagal modulation and the use of LHR as the index of sympathetic modulation might be doubtful in patients 1–2 years after OHT, because a higher HR is supposed to be associated with a lower, rather than higher, vagal modulation. Correlation analysis showed that a larger nHFP and a smaller nVFLP were associated with a decrease in mRRI in OHT patients. This is very strange if nHFP is regarded as the indicator of vagal modulation and nVLFP as the indicator of renin-angiotensin modulation, thermoregulation and vagal withdrawal. An increased vagal modulation and decreased vagal withdrawal are expected to lead to a prolonged mRRI, rather than decreased mRRI. This peculiar relation between mRRI and nHFP or nVLFP also suggested that the interpretation of nHFP as the index of vagal modulation and nVLFP as the index of renin-angiotensin modulation, thermoregulation and vagal withdrawal must be careful in OHT patients 1–2 years after surgery. Other kinds of unknown mechanism might have contributed to the increase in nHFP and the decrease in nVLFP in OHT patients, as compared with the CABG patients.

Lai et al. [30] indicated that OHT recipients’ HRV was significantly lower than that of healthy adults in terms of mean, SD_RR_, total power, low frequency, low frequency (nu), high frequency and low frequency/high frequency. In this study we found similarly that the HRV of OHT patients was severely decreased, as compared to that of CABG patients. Although the HFP of the OHT patients was significantly smaller than that of CABG patients, the nHFP in the OHT patients was greater than that of CABG patients in this study. The seemingly strange finding of increased nHFP in patients after OHT might be caused by the severely depressed VLF band relative to other frequency bands in the power spectrum. The finding of suppressed VLF band in patients after OHT seemed to be in accordance with the findings of Hadase et al. [31] have shown that the VLFP is a marker of poor recovery of autonomic control of HR in patients with congestive heart failure because a small VLFP is associated with a decreased event-free rate.

The mechanisms responsible for the loss of HRV 1–2 years after OHT might be multiple. First, the procedure of denervation after OHT should have sacrificed the intricate network of neural inter-organ connections and feedback mechanisms, producing a state of loss of complexity in the HR control system. Second, the medication used by the patients following OHT might affect the HRV of the patients. For instance, Schneider et al. [32] have shown that fetuses exposed to betamethasone in utero have an acute shift in the sympatho-vagal balance toward sympathetic suppression. Therapondos et al. [33] have reported that the HRV would be worsened by the azathioprine and prednisolone used in immunosuppression in cirrhosis patients who had received orthotopic liver transplantation.

The origin of power law characteristics has been speculated to be a manifestation of complex interaction between the heart and the autonomic nervous system. Bigger et al. [8] have shown that the power law regression parameters are excellent predictors of death of any cause or arrhythmic death and predict these outcomes better than traditional power spectral bands, and that myocardial infarction or denervation of the heart causes a steeper slope and decreased height of the power law regression relation between log(power) and log(frequency) of RR-interval fluctuations. Huikuri et al. [34] have shown that the power-law relationship of 24-h HR variability is a more powerful predictor of death than the traditional risk markers in elderly subjects, and that altered long-term behavior of HR implies an increased risk of vascular causes of death rather than being a marker of any disease or frailty leading to death. The presence of a positive slope in OHT patients instead of a negative one in many kinds of diseases [24–29] seems to echo the assertion of those studies that power law regression parameters are excellent predictors of diseases than the traditional power spectral bands parameters. From Fig. 2, it is speculated that the positive value for the slope of the power law relation of HRV might be caused by the suppression of the power in the VLF range (0.04–0.01 Hz) and the relative enhancement of the powers in the HF range in OHT patients.

Discontinuation of autonomic blocking agents such as beta-blockers for at least 24 h prior to ECG recording was requested if clinical condition allowed. However, if the medication was still needed, the discontinuation of the medications before and during the study was not considered because it was not ethical to risk the patients of possible episodes of heart attack or heart decompensation. Thus, some patients in both OHT and CABG groups were still using beta-blockers that may have effect on the HRV. Though beta-blockers can affect HRV, the use of beta-blockers between these two groups of patients was not statistically significant. Therefore, the influence of this confounding factor can be neglected in this study.

All OHT patients in this study were given immunosuppresants and prednisolone to suppress possible rejection reaction. It is therefore interesting to know whether there is a relationship between immunosuppression and sympathetic control through variations in the inflammatory state of the patient. In this study, it is impossible to compare the difference in the indices of sympathetic control between those OHT patients who have been given immunosuppresants and those patients who haven’t. However, from the literature, we know that cyclosporine-induced hypertension is associated with sympathetic neural activation, which may be accentuated by the cardiac denervation that results from heart transplantation [35]. In contrast, Schneider et al. [32] showed that there was an acute shift in the sympatho-vagal balance in fetuses exposed to betamethasone in utero toward sympathetic suppression. The immunosuppresants and steroid given to the OHT patients seem to have opposite effects on their HRV and hemodynamics. There might be a complex relation among the immunosuppression, steroid therapy and sympathetic control in the OHT patients. The final result of immunosuppressants and steroid on the sympathetic modulation of OHT patients remains to be determined.

Halpert et al. [20] have showed in a 10-year longitudinal follow-up study that the observed increase in TP, absolute LFP and HFP, but unchanged relative LFP and HFP after heart transplantation are compatible with partial re-innervation of the cardiac sinus node. Cornelissen et al. [21] have showed that the changes in HRV during long-term follow-up after heart transplantation are compatible with the cardiac sinus node, as has been suggested by cross-sectional studies. However, Beckers et al. [36] have showed in another 10-year follow-up study indicated that the vast majority of the heart transplantation showed no signs of reinnervation. Lovric et al. [37] also pointed that eventual sinus node sympathetic reinnervation and left ventricular sympathetic reinnervation do not occur simultaneously. Although the HRV measures may not be able to represent the autonomic modulation of the OHT patients when the re-innervation of the sinus node has not reached an appreciable degree after the surgery, the studies of Halpert et al. [20], Cornelissen et al. [21], and Beckers et al. [36] suggested that some HRV measures such as TP, LFP, and HFP may be associated with the re-innervation of the sinus node in the OHT patients. If this is true, then some HRV measures may be included in the post-transplantation rehabilitation criteria so that the progression of cardiac re-innervation and the restoration of autonomic control of the heart can be monitored serially and non-invasively.

In conclusion, patients after OHT have residual HRV which were characterized by severely depressed time and frequency domain HRV, increased HR and nHFP, decreased nVLFP, and a positive value for the slope of the power-law relation of HRV. There was a severe depression in the VLF band among various frequency bands in the power spectrum of RRI in patients after OHT. The interaction between the transplanted heart and various controlling systems might account for the residual HRV in patients after OHT. The use of nHFP as the indicators of vagal modulation and the use of nVLFP as the indicator of renin-angiotensin modulation, thermoregulation and vagal withdrawal must be careful in the OHT patients.

## References

[1] Akselrod S, Gordon D, Madwed JB, Snidman NC, Shannon DC, Cohen RJ (1985). Hemodynamic regulation: investigation by spectral analysis. Am J Physiol.

[2] Pomeranz B, Macaulay RJB, Caudill MA, Kutz I, Adam D, Gordon D, Kilborn KM, Barger AC, Shannon DC, Cohen RJ, Benson H (1985). Assessment of autonomic function in humans by heart rate spectral analysis. Am J Physiol.

[3] Malliani A, Lombardi F, Pagani M (1994). Power spectrum analysis of heart rate variability: a tool to explore neural regulatory mechanisms. Br Heart J.

[4] Saul JP, Arai Y, Berger RD, Lilly LS, Colucci WS, Cohen RJ (1988). Assessment of autonomic regulation in chronic congestive heart failure by heart rate spectral analysis. Am J Cardiol.

[5] Taylor JA, Carr DL, Myers CW, Eckberg DL (1998). Mechanisms underlying very-low-frequency RR-interval oscillations in humans. Circulation.

[6] Lu WA, Kuo CD (2003). The effect of Tai Chi Chuan on the autonomic nervous modulation in older persons. Med Sci Sports Exerc.

[7] Lu WA, Kuo CD (2003). The effect of wai tan kung on autonomic nervous modulation in the elderly. J Biomed Sci.

[8] Bigger JT, Steinman RC, Rolnitzky LM, Fleiss JL, Albrecht P, Cohen RJ (1996). Power law behavior of RR-interval variability in healthy middle-aged persons, patients with recent acute myocardial infarction, and patients with heart transplants. Circulation.

[9] Akselrod S, Gordon D, Ubel FA, Shannon DC, Barger AC, Cohen RJ (1981). Power spectrum analysis of heart rate fluctuation: a quantitative probe of beat-to-beat cardiovascular control. Science.

[10] Spinelli L, Petretta M, Marciano F, Testa G, Rao MA, Volpe M, Bonaduce D. Cardic autonomic responses to volume overload in normal subjects and in patients with dilated cardiomyopathy. Am J Physiol. 1999;277:H1361–8. 10.1152/ajpheart.1999.277.4.H1361 10516170

[11] Pagani M, Lombardi F, Guzzetti S, Rimoldi O, Furlan R, Pizzinelli P, Sandrone G, Malfatto G, Dell’Orto S, Piccaluga E, Turiel M, Baselli G, Cerutti S, Malliani A (1986). Power spectral analysis of heart rate and arterial pressure variabilities as a marker of sympatho-vagal interaction in man and conscious dog. Circ Res.

[12] Smith ML, Ellenbogen KA, Eckberg DL, Sheehan HM, Thames MD (1990). Subnormal parasympathetic activity after transplantation. Am J Cardiol.

[13] Ryan C, Hollenberg M, Harvey DB, Gwynn R (1976). Impaired parasympathetic responses in patients after myocardial infarction. Am J Cardiol.

[14] Tristani FE, Kamper DG, McDermott DJ, Peters BJ, Smith JJ (1977). Alternations of postural and Valsalva responses in coronary heart disease. Am J Physiol.

[15] Hayano J, Sakakibara Y, Yamada M, Ohte N, Fujinami T, Yokoyama K, Watanabe Y, Takata K (1990). Decreased magnitude of heart rate spectral components in coronary artery disease: its relation to angiographic severity. Circulation.

[16] Willman VL, Cooper T, Hanlon CR (1964). Return of neural responses after autotransplantation of the heart. Am J Physiol.

[17] Kaye MP, Randall WC, Hageman GR, Geis WP, Priol DV (1977). Chronology and mode of reinnervation of the surgically denervated canine heart: functional and chemical correlates. Am J Physiol.

[18] Kontos HA, Thames MD, Lower RR (1970). Responses to electrical and reflex autonomic stimulation in dogs with cardiac transplantation before and after reinnervation. J Thorac Cardiovasc Surg.

[19] Bristow M (1990). The surgically denervated, transplanted heart. Circulation.

[20] Halpert I, Goldberg AD, Levine AB, Levine TB, Kornberg R, Kelly C, Lesch M (1996). Reinnervation of the human heart as evidenced from heart rate variability studies. Am J Cardiol.

[21] Cornelissen VA, Vanhaecke J, Aubert AE, Fagard RH (2012). Heart rate variability after heart transplantation: a 10-year longitudinal follow-up study. J Cardiol.

[22] Task Force of the European Society of Cardiology and the North American Society of Pacing and Electrophysiology (1996). Heart rate variability: standards of measurement, physiological interpretation, and clinical use. Circulation.

[23] Malliani A, Pagani M, Lombardi F, Cerutti S (1991). Cardiovascular neural regulation explored in the frequency domain. Circulation.

[24] Tibby SM, Frndova H, Durward A, Cox PN (2003). Novel method to quantify loss of heart rate variability in pediatric multiple organ failure. Crit Care Med.

[25] Ribeiro AL, Lombardi F, Sousa MR, Lins Barros MV, Porta A, Costa Val Barros V, Gomes ME, Santana Machado F, Otávio Costa Rocha M (2002). Power-law behavior of heart rate variability in Chagas’ disease. Am J Cardiol.

[26] Jokinen V, Syvanne M, Makikallio TH, Airaksinen KE, Huikuri HV (2001). Temporal age-related changes in spectral, fractal and complexity characteristics of heart rate variability. Clin Physiol.

[27] Lin LY, Lin JL, Du CC, Lai LP, Tseng YZ, Huang SK (2001). Reversal of deteriorated fractal behavior of heart rate variability by beta-blocker therapy in patients with advanced congestive heart failure. J Cardiovasc Electrophysiol.

[28] Lombardi F, Porta A, Marzegalli M, Favale S, Santini M, Vincenti A, De Rosa A (2000). Implantable Cardioverter Defibrillator-Heart Rate Variability Italian Study Group. Implantable Cardioverter Defibrillator-Heart Rate Variability Italian Study Group. Heart rate variability patterns before ventricular tachycardia onset in patients with an implantable cardioverter defibrillator. Participating Investigators of ICD-HRV Italian Study Group. Am J Cardiol.

[29] Kucera JP, Heuschkel MO, Renaud P, Rohr S (2000). Power-law behavior of beat-rate variability in monolayer cultures of neonatal rat ventricular myocytes. Circ Res.

[30] Lai FC, Chang WL, Jeng C (2012). The relationship between physical activity and heart rate variability in orthotopic heart transplant recipients. J Clin Nurs.

[31] Hadase M, Azuma A, Zen K, Asada S, Kawasaki T, Kamitani T, Kawasaki S, Sugihara H, Matsubara H (2004). Very low frequency power of heart rate variability is a powerful predictor of clinical prognosis in patients with congestive heart failure. Circ J.

[32] Schneider U, Fiedler A, Schröder B, Jaekel S, Stacke A, Hoyer D, Schleussner E (2010). The effect of antenatal steroid treatment on fetal autonomic heart rate regulation revealed by fetal magnetocardiography (fMCG). Early Hum Dev.

[33] Therapondos G, Flapan AD, Dollinger MM, Garden OJ, Plevris JN, Hayes PC (2002). Cardiac function after orthotopic liver transplantation and the effects of immunosuppression: a prospective randomized trial comparing cyclosporin (Neoral) and tacrolimus. Liver Transpl.

[34] Huikuri HV, Mäkikallio TH, Airaksinen KE, Seppänen T, Puukka P, Räihä IJ, Sourander LB (1998). Power-law relationship of heart rate variability as a predictor of mortality in the elderly. Circulation.

[35] Scherrer U, Vissing SF, Morgan BJ, Rollins JA, Tindall RS, Ring S, Hanson P, Mohanty PK, Victor RG (1990). Cyclosporine-induced sympathetic activation and hypertension after heart transplantation. N Engl J Med.

[36] Beckers F, Ramaekers D, Speijer G, Ector H, Vanhaecke J, Verheyden B, Van Cleemput J, Droogné W, Van de Werf F, Aubert AE (2004). Different evolutions in heart rate variability after heart transplantation: 10-year follow-up. Transplantation.

[37] Lovric SS, Avbelj V, Trobec R, Zorman D, Rakovec P, Hojker S, Gersak B, Milcinski M (2004). Sympathetic reinnervation after heart transplantation, assessed by iodine-123 metaiodobenzylguanidine imaging, and heart rate variability. Eur J Cardiothorac Surg.

